# The Lipid Composition of Serum-Derived Small Extracellular Vesicles in Participants of a Lung Cancer Screening Study

**DOI:** 10.3390/cancers13143414

**Published:** 2021-07-08

**Authors:** Mateusz Smolarz, Agata Kurczyk, Karol Jelonek, Joanna Żyła, Łukasz Mielańczyk, Magdalena Sitkiewicz, Monika Pietrowska, Joanna Polańska, Witold Rzyman, Piotr Widłak

**Affiliations:** 1 Maria Sklodowska-Curie National Research Institute of Oncology, Gliwice Branch, 44-102 Gliwice, Poland; mateusz.smolarz@io.gliwice.pl (M.S.); agata.kurczyk@io.gliwice.pl (A.K.); karol.jelonek@io.gliwice.pl (K.J.); monika.pietrowska@io.gliwice.pl (M.P.); 2 Department of Data Science and Engineering, Silesian University of Technology, 44-100 Gliwice, Poland; joanna.zyla@polsl.pl (J.Ż.); joanna.polanska@polsl.pl (J.P.); 3 Department of Histology and Cell Pathology, Faculty of Medical Sciences in Zabrze, Medical University of Silesia, 40-055 Katowice, Poland; lmielanczyk@sum.edu.pl; 4 Thoracic Surgery Department, Medical University of Gdansk, 80-210 Gdansk, Poland; magdalena.sitkiewicz@gumed.edu.pl (M.S.); wrzyman@gumed.edu.pl (W.R.)

**Keywords:** biomarkers, early detection, exosomes, extracellular vesicles, lung cancer, metabolomics, screening, serum

## Abstract

**Simple Summary:**

Molecular components of extracellular vesicles present in serum are potential biomarkers of lung cancer, however, none of them have been validated in the context of an actual early detection of lung cancer. Here, we compared the lipid profiles of vesicles obtained from participants in a lung cancer screening study, including patients with screening-detected cancer and individuals with benign pulmonary nodules or without pathological changes. A few lipids whose levels were different between compared groups were detected, including ceramide Cer(42:1) upregulated in vesicles from cancer patients. Furthermore, a high heterogeneity of lipid profiles of extracellular vesicles was observed, which impaired the performance of classification models based on specific compounds.

**Abstract:**

Molecular components of exosomes and other classes of small extracellular vesicles (sEV) present in human biofluids are potential biomarkers with possible applicability in the early detection of lung cancer. Here, we compared the lipid profiles of serum-derived sEV from three groups of lung cancer screening participants: individuals without pulmonary alterations, individuals with benign lung nodules, and patients with screening-detected lung cancer (81 individuals in each group). Extracellular vesicles and particles were purified from serum by size-exclusion chromatography, and a fraction enriched in sEV and depleted of low-density lipoproteins (LDLs) was selected (similar sized vesicles was observed in all groups: 70–100 nm). The targeted mass-spectrometry-based approach enabled the detection of 352 lipids, including 201 compounds used in quantitative analyses. A few compounds, exemplified by Cer(42:1), i.e., a ceramide whose increased plasma/serum level was reported in different pathological conditions, were upregulated in vesicles from cancer patients. On the other hand, the contribution of phosphatidylcholines with poly-unsaturated acyl chains was reduced in vesicles from lung cancer patients. Cancer-related features detected in serum-derived sEV were different than those of the corresponding whole serum. A high heterogeneity of lipid profiles of sEV was observed, which markedly impaired the performance of classification models based on specific compounds (the three-state classifiers showed an average AUC = 0.65 and 0.58 in the training and test subsets, respectively).

## 1. Introduction

Lung cancer is the leading cause of cancer mortality, responsible for about one-fifth of cancer-related deaths worldwide. The majority of lung cancer cases are diagnosed at advanced stages and have a poor prognosis (average 5-year survival of about 10–15%). However, in the case of diseases detected at early stages, the prognosis is much better (the average 5-year survival in the range of 65–85%). Thus, in addition to primary prevention (i.e., tobacco smoking control), screening for early detection of lung cancer might be a major strategy to reduce lung cancer mortality [1,2]. Nowadays, low dose computed tomography (LDCT) screening in high-risk groups is the most efficacious strategy for lung screening, with a perspective for worldwide cancer mortality reduction [3,4]. However, the relatively low positive predictive value and the high percentage of false positive results of this test may lead to “over-diagnosis”, resulting in further costly, unnecessary, and potentially risky diagnostic workup [5]. Therefore, complementation of LDCT-based imaging with other clinical or molecular tests allowing for effective and reliable pre-selection of individuals for LDCT examination, or for better discrimination between benign and malignant nodules detected by LDCT, seems to be a critical issue for the practical application of the lung cancer screening strategy [6].

The overall response of human organisms to pathological conditions is mirrored in different molecular fractions of body fluids. Blood is the most widely available source of biomarkers potentially enhancing the power of early lung cancer detection or discrimination of lung nodules. Several components of blood, including circulating tumor cells, circulating tumor DNA, microRNA, autoantibodies, and specific serum/plasma proteins, have been analyzed in the search for such biomarkers [7,8,9,10], but none have been widely adopted in clinics yet. Moreover, serum metabolome represents a novel potential source of cancer markers, and a few large studies revealed sets of metabolites whose serum/plasma levels discriminated lung cancer patients from matched controls [11,12,13]. However, multiple lifestyle-related and clinical factors increase the heterogeneity of serum metabolomes [14,15], which could reduce the possibility of finding signatures that differentiate patients with low advanced lung cancer from other participants of a lung cancer screening if the whole serum metabolome is addressed [16].

Extracellular vesicles circulating in human biofluids, a potential source of cancer biomarkers, appeared recently as an attractive type of “liquid biopsy”. Small extracellular vesicles (sEV), including endosome-derived exosomes, are virus-sized (30–150 nm) nanovesicles released by many cell types in both physiological and pathological conditions. These vesicles contain a wide variety of components such as coding and non-coding RNAs, proteins, and lipids forming their bilayer membrane, which, altogether, reflect the molecular composition of parental cells. Small extracellular vesicles are involved in many aspects of cell-to-cell communication, including the crosstalk between cancer, its microenvironment, and the immune system [17,18,19]. Tumor-derived exosomes (TEX) appear to be valuable non-invasive surrogates for cancer tissue, yet their purification from other fractions of sEV circulating in patients' blood remains a major challenge. Hence, the overall serum/plasma sEV load remains a practical and potentially useful source of cancer biomarkers [20]. The diagnostic potential of serum sEV in lung cancer has been tested in several papers, mostly focused on their miRNA [21] and protein [22,23,24] cargo. However, none of the reported studies were based on material collected prospectively in the frame of a screening study; hence, the relevance of the resulting sEV-based molecular signatures for the early detection of lung cancer remains unclear.

Here, we aimed to evaluate the potential biomarker value of overall serum sEV for the detection of lung cancer or discrimination between malignant and benign lung nodules based on material collected solely from high-risk participants of a prospective lung cancer screening study (pre-diagnostic samples). Lipid components of serum sEV analyzed quantitatively by a mass-spectrometry-based approach were addressed in particular.

## 2. Materials and Methods

### 2.1. Study Subject

Material for this study was collected prospectively during the MOLTEST-BIS Lung Cancer Screening Study performed by Gdansk Medical University between 2016 and 2018. This program offered LDCT examinations for current or former smokers with at least a 20 pack-year history, aged from 50 to 75 years. This report involves three groups of participants of the MOLTES-BIS study (81 individuals in each group): (i) patients who were finally diagnosed with lung cancer, (ii) participants with CT-detected lung nodules that were confirmed to be benign by histopathology, and (iii) participants with no CT-detected lung nodules that have no other cancer-related health problem (this represented cancer cases diagnosed among 4500 participants of the screening and corresponding controls). Groups were matched according to age and smoking history; Table 1 shows the characteristics of all groups. The study was approved by the institutional Ethics Committees (Gdańsk Medical University, approval no. NKBBN/376/2014; and MSC National Research Institute of Oncology, approval no. KB/430–21/20), and all participants provided informed consent indicating their voluntary participation in the project and provision of blood samples for future research.

### 2.2. Extracellular Vesicle Preparation

Peripheral blood was collected into a 5-milliliter BD Vacutainer Tube, incubated for 30 min at room temperature to allow clotting, and then centrifuged at 1000× *g* for 10 min to remove the clot. The serum was aliquoted and stored at −80 °C before further processing. Extracellular vesicles were isolated by micro-SEC (size-exclusion chromatography) from 0.84 mL of serum in each case. Serum was pre-purified by a series of centrifugations at 1000 and 10,000× *g* for 10 and 30 min at 4 °C, respectively; then, the supernatant was filtrated using a 0.22-micrometer syringe filter unit (Roth, Karlsruhe, Germany; PA49.1). Phosphate-buffered saline (PBS) was added to the filtered serum to equilibrate its final volume to 1 mL; then, the sample was loaded onto an Econo-Pac 10DG column (BioRad, Hercules, CA, USA; 732-2010) filled with 10 mL of Sepharose CL-2B (GE Healthcare, Chicago, IL, USA; 17014001) at 6 cm length. The column was left until dripping ceased (void volume); then, the first 1 mL fraction was eluted by loading 1 mL of PBS. Further fractions were eluted analogously; sEV were eluted in fractions 3 and 4 (F3 and F4). For further lipidomics analysis, 950 µL of fraction F3 was concentrated to 50 µL using Vivaspin500 ultrafiltration tubes (Sartorius, Göttingen, Germany; VS0102) according to the manufacturer's instructions.

### 2.3. Vesicle Characterization

The presence of sEV in the collected SEC fractions was monitored in representative samples by Western blot using typical exosome markers (CD9, CD63, CD81, TSG101, and FLOT1). Moreover, proteins characteristic for other cellular components (GRP94, GM130, CD35, PHB1, and APOB) were analyzed in the same samples. Proteins were analyzed by Western blot using either 20 µL of the non-concentrated SEC fraction or an amount corresponding to 0.65 µg of the proteins (the concentration of proteins in the analyzed samples was assessed using the PierceTM BCA Protein Assay kit (Thermo Fisher Scientific, Waltham, MA, USA; 23225)). Western blot analysis was performed as described in detail elsewhere [25]. The size distribution profile of particles present in the fraction F3 from all included samples was analyzed by dynamic light scattering (DLS) using a Zetasizer Nano-ZS90 instrument (Malvern Instruments, Malvern, UK). Briefly, 50 μL of the non-concentrated fraction F3 was analyzed at 20 °C immediately after isolation in disposable low-volume cuvettes (Malvern Instruments, Malvern, UK; ZEN0118). The dispersant refractive index was 1.330 (ICN PBS Tablets) and the equilibration time was set to 30 s. The results were the average of 5 measurements consisting of 10 runs (Malvern Zetasizer Software 7.12 was used for analysis; Malvern Instruments, Malvern, UK). To perform sEV imaging by transmission electron microscopy (TEM), 1000 µL of the representative fractions F3 and F4 was concentrated to 50 µL as described above; then, equal volumes of the sEV sample and 4% paraformaldehyde (PFA) were mixed and incubated at 4 °C. The TEM analysis was performed according to the protocol provided by Thery et al. [26] as described in detail elsewhere [25].

### 2.4. Targeted Metabolomics

Briefly, 50 µL of concentrated sEV sample was obtained (the amount corresponding to approximately 800 µL of serum in each case); samples were further concentrated to 20 µL by vacuum-drying. The metabolome of sEV was analyzed by a targeted quantitative approach using the Absolute IDQ p400 HR kit (Biocrates Life Sciences, AG, Innsbruck, Austria; 96-well format) according to the manufacturer’s protocol (Supplementary Materials). This is a commercial assay kit with an automated workflow, whose quality, stability, and repeatability were validated in the ring trial [27]. This strategy hypothetically allows simultaneous quantification of 408 metabolites or their isomer groups: 42 amino acids and biogenic amines, 55 acylcarnitines, 60 di/triglycerides, 196 (lyso)phosphatidylcholines, 40 sphingolipids, 14 cholesteryl esters, and hexose. Lipids and hexoses were measured by flow injection analysis (FIA-MS) and small molecules were measured by liquid-chromatography–mass-spectrometry (LC-MS). Briefly, a 96-well-based sample preparation device was used that consists of inserts that have been impregnated with internal standards; a predefined sample amount was added to the insert, and then the target analytes were extracted with an organic solvent. High-resolution mass spectrometry (HRMS) analyses were carried out on an Orbitrap Q Exactive Plus mass spectrometer (Thermo Fisher Scientific, Waltham, MA, USA) equipped with a 1290 Infinity UHPLC (Agilent, Santa Clara, CA, USA) system using an Agilent Zorbax Eclipse XDB-C18 (3.5 μm) 3.0 × 100 mm column and controlled by Xcalibur 4.1. software. The acquired data were processed using Xcalibur 4.1. (Thermo Fisher Scientific) and MetIDQ DB110-2976 (Biocrates Life Sciences) software. The concentrations of all metabolites were quantitated in μmol. The same analysis was performed using non-fractionated whole serum (10 µL samples).

### 2.5. Data Normalization

Two types of “missing” values were detected in the metabolomics dataset: “zero” values below the level of detection/quantitation, and measurements lacking due to the internal standard error. Fifty and ten percent of missing values in each patient group were allowed for either type of error, respectively, as recommended in [28]; otherwise, the compound was excluded from further analyses. The final dataset comprised 352 metabolites where missing values were imputed. “Zero” values were replaced with random numbers generated from normal distribution truncated to a segment between 0 and the limit of quantitation for a given test plate. Lacking measurements were filled by values imputed using the k-nearest neighbor approach; the nearest observed data were identified using a correlation distance metric, and the mean value of the three nearest neighbors was used (based on measurements collected for the same patient group using the same test plate). After imputation of the missing values, the dataset was batch-corrected using an empirical Bayes method [29]; we assumed that samples measured using one 96-well sample preparation plate represent one batch. Before batch adjustment, data were transformed using the log base 2 function. The batch-corrected data were analyzed using the non-linear dimension reduction algorithm (UMAP) for the dataset structure visualization [30].

### 2.6. Statistical Analyses

Differences in the average and the most frequent sEV size were analyzed using the Kruskal–Wallis test. Furthermore, the two-sample Kolmogorov–Smirnov test was applied for pairwise comparisons of the distribution of vesicle sizes. To analyze the levels of metabolites used in quantitative analyses (201 compounds with less than 50% initial “zero” values in each group), the Kruskal–Wallis test was applied followed by the post hoc Conover test for pairwise comparisons. Moreover, the chi-squared independence test was applied to test whether the absence/presence status of the remaining 151 compounds was a group-related feature. The Benjamini–Hochberg procedure for multiple testing correction was applied when necessary. All statistical hypotheses were tested at the 5% significance level. Moreover, the eta-squared effect size was calculated for Kruskal–Wallis, whereas the Conover test statistic was standardized by the square root of the sample’s size, whose interpretation corresponds to the Pallant “r” effect size. The classification model was constructed using the multinomial logistic regression (MLR) approach. Furthermore, 8-fold cross-validation was applied. At each fold (iteration), eight samples were extracted from each group as a test set; then, the internal multiple random cross-validation (MRCV) procedure was applied for the remaining samples (training set) after splitting the data into teaching (70%) and testing (30%) subsets. Then, a forward feature selection for MLR on the teaching subset with the stop criterion ΔBIC ≤ 2 was applied, followed by classification of predicted probability to one of three groups by maximum a posteriori and evaluation of accuracy on the teaching and testing subsets. The MRCV procedure was repeated 100 times, and the resulting models were used for feature ranking generation. The features (i.e., metabolites) included in each model were sorted by their order of addition in the forward procedure; then, the elbow technique was used to extract the most relevant compound for the final MLR model. The classification parameters (the overall accuracy, AUC, sensitivity, and specificity) were evaluated in the training and test sets for each of the 8 folds (iterations) of the procedure, and the mean values were calculated with a 95% confidence interval (CI). The final model was built using the elbow technique on summary ranking from 8-fold cross-validation. Two-class models were constructed using the same pipeline; however, a model threshold was set using Youden’s J statistic.

## 3. Results

**Figure 1:**
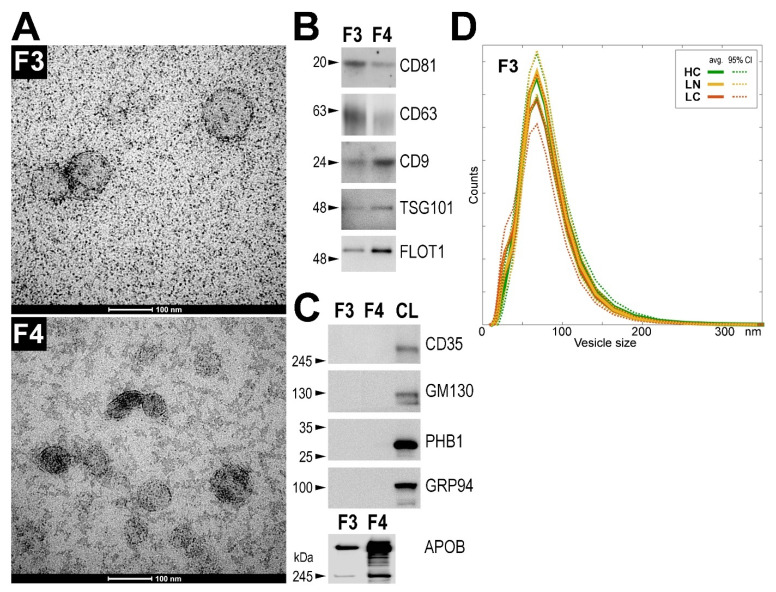
Characteristics of serum-derived sEV. (**A**) TEM imaging of vesicles present in fractions F3 and F4 (scale bars represent 100 nm); (**B**) presence of exosome markers and (**C**) markers of other cellular components detected by Western blot in sEV fractions F3 and F4; CL—positive control corresponding to whole cell lysates of human HCT116 cell. (**D**) Distribution of sizes of vesicles in sEV fraction F3 assessed by DLS measurement; average values (with 95% CI) in three groups of donors (healthy controls, HC; donors with benign lung nodules, LN; lung cancer patients, LC) are presented. Raw Western blot images are available in Figure S1.

We focused on two fractions of serum sEV separated by size-exclusion chromatography. The first EV-containing fraction (fraction F3) contained mainly vesicles of an average size of about 70–100 nm as determined by transmission electron microscopy (TEM) (Figure 1A, upper panel). The results of TEM imaging of the fraction F3 were coherent with the dynamic light scattering (DLS) analysis, which revealed an average vesicle size of about 70–80 nm. The subsequent SEC fraction F4, in addition to the vesicles of an average size of 70–100 nm, contained large amounts of smaller particles (<30 nm) (Figure 1A, bottom panel). Both fractions F3 and F4 contained exosome biomarkers (CD9, CD63, CD81, TSG101, and FLOT1) (Figure 1B) but were depleted of proteins typical for the endoplasmic reticulum (GRP94), Golgi apparatus (GM130), cell membrane (CD35), or mitochondria (PHB1) (Figure 1C). Although an amount of APOB, a protein characteristic for serum LDL and VLDL particles, was detected in fraction F3, a much higher amount of this protein was observed in fraction F4 (Figure 1C). This was in agreement with a large number of particles present in fraction F4 that corresponded in size with LDL bodies (i.e., 22–27 nm). Therefore, despite the larger amount of material present in fraction F4, we selected fraction F3 for further analysis to reduce the influence of lipoproteins putatively present in the subsequent fractions.

Three matched groups of donors were compared: patients with screening-detected lung cancer (LC), individuals with benign lung nodules (LN), and individuals with no lung pulmonary alterations (healthy controls, HC), with 81 patients in each group (groups’ characteristics are shown in Table 1). The size distribution of the serum-derived sEV was compared in these groups of donors using the DLS approach, and no statistically significant difference was noted between groups. The distribution of the sEV sizes in fraction F3 (in the 40–120 nm range) was similar in all three groups, and the average (the most frequent) diameter of the vesicles was about 70 nm in each case according to the DLS-based estimation (Figure 1D). Similarly, no statistically significant differences between groups were noted when the sizes of vesicles present in fraction F4 were compared.

**Figure 2:**
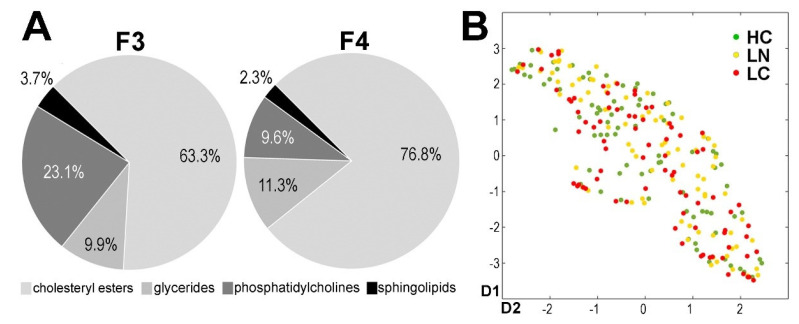
Characteristics of lipid profiles in sEV. (**A**) Relative contribution of major classes of lipids in sEV fractions F3 and F4 (polled samples obtained from sera of 4 healthy donors were analyzed); (**B**) the global structure of the whole sEV lipid dataset. A spatial visualization was created using the UMAP data transformation from 352-dimensional metabolic space to 2D view, preserving the structure of the high-dimensional data to illustrate the potential sample clustering (HC—healthy controls; LN—benign lung nodules; LC—lung cancer).

The metabolite profile of serum sEV was analyzed using the mass-spectrometry-based approach, which enabled targeted quantitative analysis of about 400 compounds (or isomer groups). However, the levels of the majority of amino acids and biogenic amines that could hypothetically be analyzed by this test were below the limit of detection or quantitation. Therefore, these two classes of metabolites were removed from the analysis, which stayed focused on lipids. In general, cholesterol derivatives (cholesteryl esters) appeared to be the most abundant class of lipids detected in the analyzed vesicles (about two-thirds of the total lipid amount detected by the test), while choline-containing glycerophospholipids (phosphatidylcholines and lysophosphatidylcholines) comprised about a quarter of the total lipids in fraction F3. Noteworthy, in the subsequent fraction F4, the relative contribution of cholesterol derivatives increased (to approx. 77%), while the contribution of choline-containing phospholipids decreased (to approx. 10%), which putatively reflected the increased presence of cholesterol-containing LDL particles in this fraction (Figure 2A).

A whole set of lipids detected in fraction F3 was analyzed by an unsupervised approach to reveal a potential “global pattern” of variances within and between groups of donors. The data were transformed by the UMAP dimension reduction tool from 352-dimensional metabolic space to 2-dimensional projection, with preservation of the dataset structure, which allowed for illustrating the global dataset structure and, thus, potential sample clustering. It is noteworthy that we observed a high inter-individual heterogeneity of samples and no separation of the three patient groups (HC, LN, and LC), as illustrated in Figure 2B.

**Figure 3:**
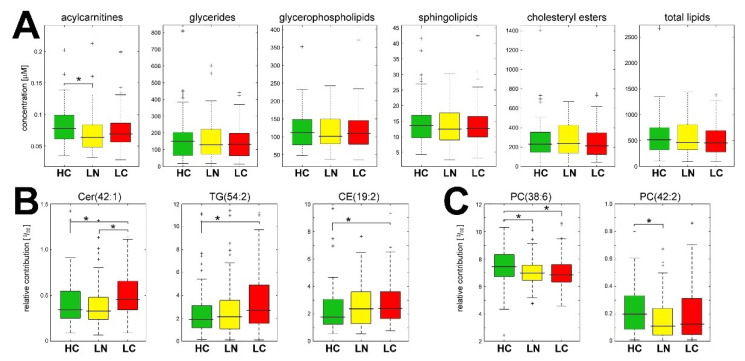
Level of lipids in serum-derived sEV. (**A**) Aggregated concentration of lipids from major classes in sEV samples; (**B**) relative contribution of selected lipids that were upregulated or (**C**) downregulated in the sEV of lung cancer patients. HC—healthy controls; LN—benign lung nodules; LC—lung cancer. Box plots show the median, minimum, maximum, lower, and upper quartile; crosses (+) represent outliers; asterisks (*) represent statistically significant differences between groups (*p* < 0.05 in the pairwise post hoc test).

The lipid profiles were compared quantitatively in all groups of donors (HC, LN, and LC) to detect specific differences between groups. In general, we detected 352 lipids. However, 151 lipids were detected in a minority of the samples (less than 50% in each group); hence, these compounds were excluded from the quantitative analyses. Nevertheless, the binary analysis revealed no statistically significant differences between groups if the absent/present status was compared. The remaining 201 lipids that were quantitated in the majority of samples were included in the quantitative analysis presented below (these compounds are listed in Table S1). First, we looked at the actual amounts of the detected lipids (i.e., their concentration in sEV-containing fraction F3, which corresponded to 0.8 mL of serum). When aggregated concentrations of compounds representing major classes of lipids were compared (all compounds included), no statistically significant difference was observed among groups, except for a slightly increased level of acylcarnitines in the healthy control group. Consequently, no differences in the total amounts (i.e., aggregated concentration) of lipids were observed in sEV preparations from different groups (Figure 3A). Therefore, we looked further at specific lipid compounds using their relative levels (concentrations) expressed as the permille (‰) of total lipid concentration. This type of normalization was chosen due to the lack of actual quantitation of the number of vesicles in each of the analyzed samples.

Eleven compounds showed significantly different levels between the compared groups (Kruskal–Wallis test *p*-value <0.05). Further pairwise comparisons (Conover test *p*-value <0.05) revealed six compounds that differentiated HC from LN, seven compounds that differentiated HC from LC, and five compounds that differentiated LN from LC (Table S1). Five compounds, namely ceramide Cer(42:1), triglyceride TG(54:2), and phosphatidylcholines PC(24:0), PC-O(32:0), and LPC(20:1), showed elevated levels in LC samples compared to both HC and LN samples; the relative levels of the former two compounds are illustrated in Figure 3B. Moreover, cholesteryl ester CE(19:2) showed elevated levels in both LC and LN compared to HC (also illustrated in Figure 3B). On the other hand, “classical” phosphatidylcholines with unsaturated acyl chains, namely PC(38:6), PC(42:2), PC(36:5), and PC(38:7), showed higher levels in sEV samples of HC donors compared to LC and LN donors; the relative levels of the former two compounds are illustrated in Figure 3C.

Furthermore, we aimed to assess the performance of a hypothetical biomarker based on the lipids present in serum-derived sEV. The set of 201 quantitated lipids was used to test the multi-component signature based on the relative contribution of specific compounds; the three-class model (HC vs. LN vs. LC) was analyzed. The training step allowed for establishing the rank of features (i.e., quantitated metabolites) that were the most important for classification; eight independent iterations of the multiple random cross-validation procedure were applied and tested. Tested signatures included from 8 to 21 components; LPC(20:1), PC(42:4), PC(24:0), AC(18:0), Cer(42:1), TG(54:2), CE(19:2), and PC(38:3) were selected in the classification model in at least 5 out 8 iterations (the first two were selected each time). The overall mean accuracy of the obtained models was 55.2% and 38.8% in the training and test sets, respectively (compared to 33.3% expected by chance in a three-class model). This corresponded to a mean AUC = 0.65 and 0.58 in the training and test sets, respectively (Table 2). The eight-fold cross-validation classification results, together with the corresponding levels of sensitivity and specificity, are presented in Table S2a. The final classification model was built using 20 features that were top-ranked in eight cross-validated models (details of this final model are described in Table S2b). Moreover, binary classification models were built to test specific pairs of study groups. Notably, these two-class models performed similarly to the three-class one: the average AUC values were in the range of 0.75–0.78 and 0.53–0.54 in the training and test sets, respectively (Table 2). The results of eight-fold cross-validation of classification together with the corresponding levels of sensitivity and specificity of these binary classifiers are presented in Table S3a. Furthermore, the final classification models built using the top-ranked features are presented in Table S3b. Hence, we concluded a rather low power of the classification models based on different signatures of lipids present in serum-derived sEV.

A quantitative analysis of metabolites was also performed using non-fractionated whole serum from the same group of donors (relative levels of different lipids are presented in Table S4). Significant differences in the relative contribution of lipids were observed between the whole serum and serum-derived sEV. In general, triglycerides were overrepresented, while acylcarnitines, lysophosphatidylcholines, sphingolipids, and cholesteryl esters were underrepresented in sEV compared to whole serum (Table S5). Furthermore, a comparison of the patient groups showed different results when the relative levels of specific lipids were analyzed in the whole serum and sEV. The five compounds that were most significantly upregulated in sEV from LC compared to sEV from HC, namely CE(19:2), Cer(42:1), TG(54:2), PC-O(33:3), and PC(24:0), were not upregulated in the whole serum of cancer patients (the two latter compounds were not quantified due to their low serum level). On the other hand, the two phosphatidylcholines that were significantly downregulated in sEV from LC, namely PC(38:6) and PC(38:5), were also downregulated in the serum of LC (Table 3). Moreover, different disease-related features were noted in sEV and total serum when the HC vs. LN and LN vs. LC groups were compared (Table S6). Hence, we concluded that disease-related features observed in the lipid profile of serum-derived sEV could be specific for these vesicles, not for the whole serum lipidome.

## 4. Discussion

Extracellular vesicles circulating in the biofluids of cancer patients, which represent a mixture of TEX and other classes of vesicles released by “normal” types of cells, are a potential source of diagnostic and prognostic biomarkers. Numerous studies tested protein and RNA components of such vesicles in the context of lung cancer [21]. Several lung cancer signatures were proposed based on proteins present in serum/plasma-derived sEV [22,23,24,31,32], and more reports focused on the miRNA cargo of serum/plasma-derived sEV. According to the current literature review, miRNA signatures of lung cancer have been reported in about 30 papers [21]. These signatures contained about 60 miRNA species overall, including 14 miRNA species that appeared in multiple signatures (exemplified by the oncomir miR-21). Moreover, a few studies reported lung cancer signatures based on long non-coding RNAs [33,34] or circular RNAs [35]. In contrast to the protein and RNA components of sEV, the metabolites present in these vesicles were rarely analyzed as cancer biomarkers [36]. A few works showed that metabolites present in urine-derived sEV were potential biomarkers of prostate cancer [37,38]. However, there is only one study where the metabolite profile of sEV purified by ultracentrifugation from the plasma of lung cancer patients (44 cases and 39 controls) was reported [39]. The diagnostic potential of lung cancer signatures based on different molecular components of serum/plasma-derived sEV was tested in part of the abovementioned studies, which resulted in AUC values that ranged between 0.70 and 0.95 [21]. However, different protocols were used in these studies for sEV purification and characterization. Furthermore, involved groups of patients and controls have different demographic/clinical characteristics and genetic backgrounds. Therefore, the biomarker potential of the proposed signatures could not be compared directly. Moreover, none of these studies included material collected prospectively in the high-risk population during the lung cancer screening study; hence, the potential of the proposed biomarkers for actual early lung cancer detection has not been addressed effectively yet.

Several studies documented that different classes of sEV isolated from human serum/plasma co-purify with apolipoprotein-containing particles such as LDL and VLDL [40,41]. Noteworthy, simple methods used typically for sEV purification such as precipitation, ultracentrifugation, and size-exclusion chromatography cannot separate exosomes/sEV from LDL/VLDLs [25,40,41]. Therefore, the combination of size-exclusion chromatography with gradient ultracentrifugation was postulated for the efficient separation of these two components [41]. However, the proposed procedure was rather laborious and required a large amount of material, which made it impractical in routine diagnostic applications. The “contamination” with LDL challenges the analysis of a proteome cargo of sEV [25]. However, lipid components of LDL/VLDLs are even more distractive when lipids present in the sEV membrane are addressed. Therefore, in our study, we decided to use the sEV-containing fraction generated by size-exclusion chromatography that was depleted of particles corresponding to apolipoprotein-containing LDL. As a consequence, the relative amounts of phosphatidylcholines and sphingolipids (i.e., typical components of plasma membrane) increased while the contribution of cholesterols (i.e., major components of LDL) decreased in the analyzed lipid profiles.

We observed that the levels of several lipids, putative components of sEV’s membranes, were different between vesicles purified from the sera of high-risk smokers participating in the lung cancer screening and those from the sera of patients with screening-detected lung cancer. Among a few compounds upregulated in vesicles from cancer patients was ceramide Cer(42:1) (a compound that has a sphingosine backbone with 18 carbons and a fatty acyl chain with 24 carbons). In general, ceramides are important signaling molecules involved in several human pathologies including cancer. Deregulated metabolism of ceramides with long fatty acyl chains (synthesized by ceramide synthases 2) and an elevated level of Cer(42:1) were reported in colorectal tumors [42]. Furthermore, the level of Cer(42:1) increased in the sera of patients with colorectal cancer [43] and the plasma of patients with ovarian cancer [44]. Moreover, an elevated level of Cer(42:1) was reported in serum/plasma of patients with other metabolic, autoimmune, and neurodegenerative diseases, pointing to the biomarker potential of this compound [45]. However, this is the first report where this potential biomarker was revealed as a component of sEV (noteworthy, in our model, the cancer-related elevation of this compound was observed only in sEV). On the other hand, among the lipid components of sEV downregulated in vesicles from cancer patients, prevailed phosphatidylcholines with long polyunsaturated fatty acyl chains. It is noteworthy that a similar observation was reported by Fan and coworkers [39], who analyzed the lipid composition of sEV isolated by ultracentrifugation from the plasma of lung cancer patients, and by Clos-Garcia and coworkers [38], who analyzed the lipid composition of sEV isolated by ultracentrifugation from the urine of prostate cancer patients. Moreover, the compound that was most significantly downregulated in our study, i.e., PC(38:6), was also reported in both of the abovementioned studies among the significantly downregulated compounds in sEV from patients with lung cancer and prostate cancer. Therefore, the reduced contribution of phosphatidylcholines with long polyunsaturated fatty acyl chains, exemplified by PC(38:6), could be a general feature of sEV present in the body fluids of cancer patients.

We found a few lipids whose levels in serum-derived sEV were different between the three groups of participants in the lung cancer screening study, including high-risk smokers with no pulmonary lesions detected, participants with benign lung nodules, and participants ultimately diagnosed with lung cancer. Interestingly, the entire lipid composition and its important cancer-related features detected in serum-derived sEV were different than those of the corresponding whole serum. This indicated that the lipidome of serum-derived sEV is an independent source of potential biomarkers of lung cancer. However, we observed high heterogeneity of the lipid profiles of sEV within each group, which markedly impaired the performance of the classification models based on these sEV components. Similar heterogeneity was observed when whole serum metabolite profiles were analyzed in a larger group of lung cancer screening participants [16], which could be attributed to lifestyle-related factors (e.g., diet) and co-existing medical conditions [14,15]. It is noteworthy that the performance of the three-state classification model (healthy controls vs. benign nodules vs. lung cancer) based on the lipid components of serum-derived sEV was comparable to the performance of a similar classification model based on the concentration of metabolites quantitated in the whole serum (mean AUC = 0.60 with 95% CI of 0.56–0.65 in the test set) [16]. Therefore, the present study does not demonstrate the benefit of using metabolites present in the overall serum-derived sEV instead of whole-serum-derived metabolites as early biomarkers of lung cancer. However, one should be aware that hypothetical TEX released to circulation by low advanced lung cancer putatively represent a minor fraction of serum sEV, and profiling of the overall serum sEV might be not sensitive enough to detect cancer-specific features. Therefore, lipidome profiling of the serum exosomes for early detection of lung cancer might be reconsidered if feasible methods of TEX isolation become available.

## 5. Conclusions

A few lipids detected in serum-derived sEV showed different levels between samples of healthy participants in the lung cancer screening study and pre-diagnostic samples of patients with screening-detected lung cancer. This was exemplified by ceramide Cer(42:1), a compound whose increased plasma/serum level was reported in different pathological conditions, being upregulated in vesicles from cancer patients. On the other hand, the contribution of phosphatidylcholines with polyunsaturated fatty acyl chains was reduced in vesicles from lung cancer patients, which could be a general cancer-related feature of circulating sEV as it was also reported in other studies. However, a high heterogeneity of the lipid profiles of sEV was observed, which markedly impaired the performance of the classification models based on specific compounds (similarly to the high heterogeneity observed previously in whole serum metabolomes of lung cancer screening participants). Nevertheless, the data obtained do not support the concept of using the metabolites present in serum-derived “total” sEV as biomarkers for early lung cancer detection.

## Figures and Tables

**Table 1:** Characteristics of donor groups.

Group	Healthy Controls (HC)	Benign Lung Nodules (LN)	Lung Cancer Cases (LC)
Number	81	81	81
Clinical stage:	-	-	
- IA			35
- IB			6
- IIA			8
- IIB			6
- IIIA			12
- IIIB			1
- IV			13
Histopathology:	-	-	
- Adenocarcinoma			39
- Squamous cell carcinoma			24
- Not otherwise specified NSCLC			12
- SCLC			5
- Other lung cancer			1
Sex:			
- Female	34	40	35
- Male	47	41	46
Age: years (median)	54–79 (67)	51–79 (66)	54–79 (67)
Smoking: pack-year (median)	27–132 (46)	26–133 (43)	24–110 (48)

**Table 2:** Indices of the classification models.

Set/model	Three-Class Model		HC vs. LC		LN vs. LC		HC vs. LN	
	Accuracy % (95% CI)	AUC (95% CI)	Accuracy % (95% CI)	AUC (95% CI)	Accuracy % (95% CI)	AUC (95% CI)	Accuracy % (95% CI)	AUC (95% CI)
Training	55 (53–58)	0.65 (0.63–0.68)	71 (67–74)	0.76 (0.72–0.80)	70 (67–72)	0.75 (0.72–0.77)	73 (70–76)	0.78 (0.75–0.80)
Test	39 (32–46)	0.58 (0.51–0.64)	54 (42–65)	0.54 (0.38–0.70)	54 (47–62)	0.53 (0.44–0.63)	54 (46–61)	0.53 (0.46–0.61)

Shown are the overall accuracy and area under the receiver operating characteristic (AUC) for the three-class model (HC vs. LN vs. LC) and binary two-class models (mean values and 95% confidence intervals (CI)).

**Table 3:** Comparison of lipid components in sEV and whole serum. Listed are the top five compounds upregulated and downregulated in sEV from cancer patients; shown is the *p*-value for LC vs. HC pairwise comparison, the *p*-value-based rank, and the corresponding LC/HC fold-change (median-based); bold characters reflect statistical significance.

Compound	sEV (Fraction 3)			Whole Serum		
	LC vs. HC *p*-Value	Rank	LC/HC Fold-Change	LC vs. HC *p*-Value	Rank	LC/HC Fold-Change
Upregulated in sEV of cancer patients (LC)						
CE(19:2)	**0.006**	1	1.360	0.959	97	0.951
Cer(42:1)	**0.013**	2	1.326	0.367	41	1.002
TG(54:2)	**0.019**	3	1.410	0.819	79	0.926
PC-O(33:3)	**0.033**	4	1.215	Not detected/quantitated		
PC(24:0)	**0.037**	5	1.673	Not detected/quantitated		
Downregulated in sEV of cancer patients (LC)						
PC(38:6)	**0.008**	1	0.922	**0.027**	1	0.940
PC(38:7)	**0.022**	2	0.918	0.827	102	1.157
PC(37:5)	**0.024**	3	0.926	0.586	48	0.990
TG(56:8)	**0.026**	4	0.783	0.260	25	0.978
PC(38:5)	**0.027**	5	0.952	**0.048**	2	0.954

## Data Availability

The data presented in this study are available on request from the corresponding author. The data are not publicly available due to privacy and ethical reasons.

## Supplementary Materials

The following are available online at https://www.mdpi.com/article/10.3390/cancers13143414/s1, Figure S1: Raw Western blot images for Figure 1, Table S1: Lipids detected in serum-derived sEV, Table S2: Three-state classification models, Table S3: Binary classification models, Table S4: Lipids detected in whole serum, Table S5: Relative contribution of different lipids in serum-derived sEV and whole serum of cancer patients and healthy individuals, Table S6: Comparison of lipid signatures in sEV and whole serum, Supplementary File: Protocol for metabolite detection and quantification by the Absolute IDQ p400 HR kit.
