# Supplementary material for: The Lipid Composition of Serum-Derived Small Extracellular Vesicles in Participants of a Lung Cancer Screening Study
Source: Cancers (Basel). 2021 Jul 8;13(14):3414. doi: 10.3390/cancers13143414 (PMC8307680; doi:10.3390/cancers13143414)
Supplement: Supplementary file 1 [file cancers-13-03414-s001.zip › cancers-1261938-supplementary---final--/cancers-1261938-supplementary---final--.pdf]

Supplementary Materials

The Lipid Composition of Serum-Derived Small Extracellular Vesicles in Participants of a Lung Cancer Screening Study

Mateusz Smolarz, Agata Kurczyk, Karol Jelonek, Joanna Żyła, Łukasz Mielańczyk, Magdalena Sitkiewicz, Monika Pietrowska, Joanna Polańska, Witold Rzyman and Piotr Widłak

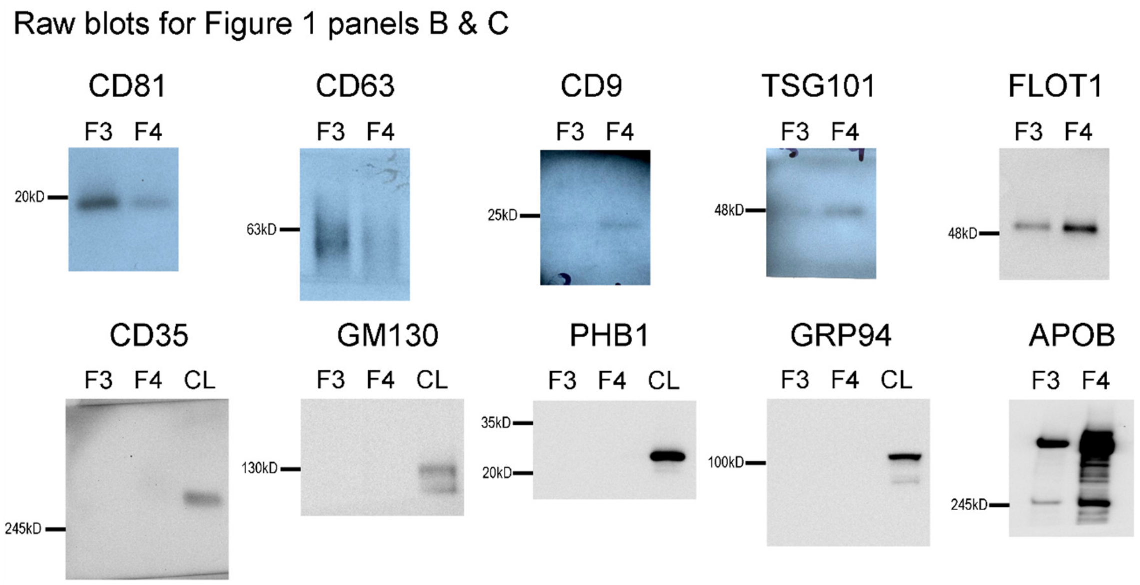

Figure S1. Raw Western blot images for Figure 1.

Supplementary File: Protocol for Metabolite Detection and Quantification by the Absolute IDQ P400 HR Kit

Samples were analyzed by a targeted quantitative approach using a combined flow injection analysis (FIA) and liquid chromatography (LC) high-resolution mass spectrometry (HRMS) assay with the Absolute IDQ p400 HR kit (Biocrates Life Sciences AG, Innsbruck, Austria). The LC-HRMS method was used to quantify amino acids and biogenic amines while acylcarnitines, cholesterol esters, glycerophospholipids, glycerides, sphingolipids, and hexoses were assessed using FIA-HRMS. The kit provided quantitative measurements for 12 acylcarnitines, 21 amino acids, 21 biogenic amines, and the sum of hexoses. For the amino acids and biogenic amines, standard solutions for calibration curves at seven levels including isotopically labeled internal standards were provided for each metabolite. For the acylcarnitines and sum of hexoses, standards at one concentration were provided (one-point calibration using FIA). The rest of the metabolites were measured semi-quantitatively, e.g., standards with similar chemical properties as the targets were used (a version of one-point calibration).

Sample preparation was performed according to the detailed protocol that was provided with the kit. Briefly, the samples were prepared on the kit plate (all plates provided with the kit were in the 96-well format) by first adding stable isotope-labeled standards followed by 50 µL of concentrated sEV sample. The samples were then dried using an evaporator and subsequently derivatized by the addition of 50 µL of a 5% solution of phenylisothiocyanate (in water:ethanol:pyridine, 1:1:1) followed by incubation at room temperature for 20 min. The samples were then dried again and extracted by the addition of 300 µL 5 mM ammonium acetate in methanol (MeOH) and shaking at 450 rpm for 30 min. The extracts were collected by centrifugation into the provided collection plate. For LC-HRMS analysis, 150 µL of the samples was transferred and diluted with 150 µL water on an empty plate, and for FIA-HRMS analysis, 250 µL of the FIA mobile phase (made by mixing 290 mL MeOH and a 10 mL ampule Biocrates FIA mobile phase additive, provided with the kit) was added directly to the samples on the collection plate.

The extracts were analyzed using a 1290 Infinity UHPLC system (Agilent, Santa Clara, CA, USA) coupled to a high-resolution Q Exactive™ plus Orbitrap mass spectrometer (Thermo Fisher Scientific, Waltham, MA, USA) using electrospray ionization. The instrumental analysis was executed according to the guidelines from the kit manufacturer. In brief, the analysis was performed in the positive ionization mode for both LC-HRMS and FIA-HRMS, and the data were collected in the full-scan (MS1) mode. The source parameters used for the LC-HRMS analysis were as follows: auxiliary gas flow rate, 30; sheath gas flow rate, 60; S-lens RF level, 60; spray voltage, 3 kV; sweep gas flow rate, 1; capillary temperature, 300 °C; auxiliary gas heater temperature, 300 °C. The parameters for FIA-HRMS were as follows: auxiliary gas flow rate, 5; sheath gas flow rate, 15; S-lens RF level, 60; spray voltage, 2.5 kV; sweep gas flow rate 1; capillary temperature, 300 °C; auxiliary gas heater temperature, 120 °C. The chromatography was executed using an Agilent Zorbax Eclipse XDB-C18 (3.5 µm) 3.0 x 100 mm column provided with the kit, with the injection volume was 5 µL. Mobile phase A was 0.2% formic acid in water and mobile phase B was 0.2% formic acid in acetonitrile. The chromatographic program lasted 6 min, including a gradient from 0 to 95% B over 4 min, followed by washing (95% B) and equilibration (0% B). The flow rate was 0.8 mL/min, and the column oven temperature was 50 °C. The FIA analysis was performed by injecting 20 µL of the sample into the flow of the FIA program, lasting for 5 min, and pumping the FIA mobile phase (10 mL ampule Biocrates FIA mobile phase additive in 290 mL MeOH) at a flow rate of 0.05 mL/min for the first 1.6 min, then increasing the flow rate to 0.2 mL/min for 1.2 min, and then changing back to 0.05 mL/min for the rest of the program.

© 2021 by the authors. Licensee MDPI, Basel, Switzerland. This article is an open access article distributed under the terms and conditions of the Creative Commons Attribution (CC BY) license (http://creativecommons.org/licenses/by/4.0/).
